# 2426 Fostering cross-disciplinary research: Lessons learned from STTEP-UP

**DOI:** 10.1017/cts.2018.212

**Published:** 2018-11-21

**Authors:** Hannibal Person, Adjoa R. Smalls-Mantey, Oluwasheyi Ayeni, Dagmar Hernandez-Saurez, Emma K. T. Benn, Emilia Bagiella, Janice L. Gabrilove

**Affiliations:** 1 Icahn School of Medicine at Mount Sinai; 2 University of Puerto Rico Medical School

## Abstract

OBJECTIVES/SPECIFIC AIMS: N/A. METHODS/STUDY POPULATION: N/A. RESULTS/ANTICIPATED RESULTS: N/A. DISCUSSION/SIGNIFICANCE OF IMPACT: There is an increasing need to foster cross-disciplinary research to address complex problems within healthcare. The Sinai Team-based Translational Education Program: the URM Propeller (STTEP-UP) is a NCATS funded program through the Icahn School of Medicine at Mount Sinai. Its goal is to facilitate URM post-doctoral trainees becoming innovative leaders in clinical and translational research. The program includes a team-based research component, where fellows collaborate on a project. This year, disciplines represented by the four fellows include Cardiology, Psychiatry, Neurology, and Pediatrics. Identifying a clinical question and designing an investigation was facilitated by group brainstorming meetings with program mentors. Fellows designed a project to identify medical testing and prescribing that were not clinically indicated throughout the healthcare system, with the goal of exploring whether an intervention, including provider education, could reduce ordering practices. In addition to regular in-person meetings, a licensed virtual learning environment and free web-based sharing platform were used to foster collaboration. Challenges faced throughout this process, included fellows struggling to find protected time, difficulties accessing broad sets of data across the healthcare system, and overcoming administrative barriers between departments. Strengths of this approach, included fellows learning new research strategies and feeling a deeper sense of commonality with their peers. Overall, this experience supports the idea that cross-disciplinary research improves the collaboration and education of emerging researchers. However, addressing logistical and systems-based barriers may better facilitate this education and research.

